# Adding dimension to the diagnostic process: Demonstration of the DSM-5 checklist and PID-5 personality trait assessment scale

**DOI:** 10.1192/j.eurpsy.2021.174

**Published:** 2021-08-13

**Authors:** L. Egervári

**Affiliations:** Department Of Psychiatry And Psychotherapy, Semmelweis University, Budapest, Hungary

**Keywords:** PID-5, Borderline personality disorder, dimensional model, categorical model

## Abstract

Borderline personality disorder (BPD) is very common, with an estimated community prevalence of 1-3%, rising to 25% in psychiatric inpatients. The aim of this talk is to address the challenges clinicians face when diagnosing borderline personality disorder. The new dimensional approach to the classification of personality disorders adopted by ICD-11 diverges from the classical categorical case definitions used in the ICD-10 and DSM-IV/DSM-5 diagnostic frameworks, thereby significantly altering the concept of personality disorders. While the DSM-5 checklist is a well-known and widely used diagnostic entity by now, with the introduction of Personality Inventory for DSM-5 (PID-5) a new assessment tool has emerged, providing the possibility of a more detailed description of personality functioning and traits. PID-5 is a 220-item self-rated personality trait assessment scale, assessing 25 personality trait facets and 5 main personality trait domains. This talk will focus on analyzing the difference between categorical and dimensional diagnostic work-up, using a case presentation to demonstrate the diagnostic processes and their outcomes.

**Disclosure:**

No significant relationships.

